# c-Jun Overexpression Accelerates Wound Healing in Diabetic Rats by Human Umbilical Cord-Derived Mesenchymal Stem Cells

**DOI:** 10.1155/2020/7430968

**Published:** 2020-01-14

**Authors:** Chun Yue, Zi Guo, Yufang Luo, Jingjing Yuan, Xinxing Wan, Zhaohui Mo

**Affiliations:** ^1^Department of Endocrinology and Metabolism, Third Xiangya Hospital of Central South University, Changsha, China; ^2^Diabetic Foot Research Center, Third Xiangya Hospital of Central South University, Changsha, China

## Abstract

**Objective:**

Mesenchymal stem cells (MSCs) are considered a promising therapy for wound healing. Here, we explored the role of c-Jun in diabetic wound healing using human umbilical cord-derived MSCs (hUC-MSCs).

**Methods:**

Freshly isolated hUC-MSCs were subjected to extensive *in vitro* subcultivation. The cell proliferative and migratory capacities were assessed by the Cell Counting Kit-8 and scratch assays, respectively. c-Jun expression was evaluated by RT-PCR and western blot analysis. The function of c-Jun was investigated with lentivirus transduction-based gene silencing and overexpression. Diabetes mellitus was induced in SD rats on a high-glucose/fat diet by streptozocin administration. Wounds were created on the dorsal skin. The effects of c-Jun silencing and overexpression on wound closure by hUC-MSCs were examined. Reepithelialization and angiogenesis were assessed by histological and immunohistochemical analysis, respectively. Platelet-derived growth factor A (PDGFA), hepatocyte growth factor (HGF), and vascular endothelial growth factor (VEGF) levels were determined by western blot analysis.

**Results:**

hUC-MSCs showed gradually decreased cell proliferation, migration, and c-Jun expression during subcultivation. c-Jun silencing inhibited cell proliferation and migration, while c-Jun overexpression enhanced proliferation but not migration. Compared with untransduced hUC-MSCs, local subcutaneous injection of c-Jun-overexpressing hUC-MSCs accelerated wound closure, enhanced angiogenesis and reepithelialization at the wound bed, and increased PDGFA and HGF levels in wound tissues.

**Conclusion:**

c-Jun overexpression promoted hUC-MSC proliferation and migration *in vitro* and accelerated diabetic wound closure, reepithelization, and angiogenesis by hUC-MSCs *in vivo*. These beneficial effects of c-Jun overexpression in diabetic wound healing by hUC-MSCs were at least partially mediated by increased PDGFA and HGF levels in wound tissues.

## 1. Introduction

Patients with diabetes mellitus (DM) often experience impaired wound healing, which leads to the formation of chronic ulcers. The underlying pathogenesis is primarily accounted for by a weakened immune system [1], insufficient angiogenesis [2], impaired proliferation and migration of keratinocytes and fibroblasts [3], and diminished production of healing-related growth factors such as vascular endothelial growth factor (VEGF) [4], insulin-like growth factor 1 (IGF-1) [5], transforming growth factor-beta (TGF-*β*) [6], and platelet-derived growth factor (PDGF) [7].

Mesenchymal stem cells (MSCs) have demonstrated great therapeutic potential for tissue repair and regeneration [8]. MSC therapy may be an especially suitable treatment for diabetic wound healing because endogenous MSCs in DM patients often exhibit low viability and impaired multiplication capacity [9]. Indeed, several recent studies have shown that the administration of MSCs improves diabetic wound healing though epithelialization, angiogenesis, and the formation of granulation tissues [6, 10, 11]. The number of MSCs isolated from tissues is often insufficient to meet the application need [12], and therefore, cells are always expanded *in vitro* following isolation to obtain sufficient amounts. However, these *in vitro* aged MSCs display reduced viability and rapid apoptosis and fail to reach the targeted wound bed after implantation, leading to diminished therapeutic effects [13, 14]. Enormous efforts have been made to improve MSC engraftment efficiency and vitality. For example, Nuschke and colleagues managed to improve MSC survival by tethering epidermal growth factor (EGF) to *β*-tricalcium phosphate bone scaffolds [15]. The administration of neurotrophin-3 promoted the secretion of VEGF, nerve growth factor (NGF), and brain-derived neurotrophic factor (BDNF) in human MSCs and thereby facilitated wound healing in diabetic mice [16]. Despite these advances, further research is required to identify new approaches to maximize MSC robustness as a therapy for diabetic wound healing.

c-Jun is an essential component of AP-1 (activator protein-1), an early response transcriptional factor that regulates the expression of a diverse range of genes [17]. AP-1 is involved in many biological processes including cell proliferation, differentiation, migration, and apoptosis, as well as inflammatory responses and tumorigenesis [18–20]. Studies have shown that c-Jun is essential for the formation of the epidermal leading edge by controlling an EGF autocrine loop and thereby plays an important role in cutaneous wound healing [21, 22]. Moreover, c-Jun-deficient fibroblasts have been reported to inhibit the proliferation of co-cultured keratinocytes [23]. However, whether c-Jun controls MSC robustness is unknown.

In the present study, we investigated the role of c-Jun in regulating the proliferation, migration, and growth factor production of cultured human umbilical cord-derived MSCs (hUC-MSCs). We found that hUC-MSCs gradually lost their proliferative and migratory capacities as well as c-Jun expression during *in vitro* expansion, and c-Jun overexpression increased hUC-MSC proliferation and growth factor production. Furthermore, hUC-MSCs overexpressing c-Jun exhibited greater efficacy in promoting wound repair in diabetic rats compared with control cells. These findings unveil a new strategy to improve the therapeutic effects of MSCs in treating diabetic wound healing.

## 2. Materials and Methods

### 2.1. Isolation and Culture of hUC-MSCs

Umbilical cords were collected from healthy donors. The protocol was approved by the Ethics Committee at the Third Xiangya Hospital of Central South University (CSU; Changsha, Hunan, China). Immediately after collection, the umbilical cords were rinsed in sterile saline, cut into 2-3 mm sections, and digested at 37°C for 4 hours in Dulbecco's modified Eagle's medium (DMEM; Gibco, USA) containing 0.1% collagenase I (Sigma-Aldrich Co., USA). The resulting cell suspension was filtered through 75 *μ*m cell strainers and centrifuged at 1,500 rpm for 10 minutes. The cells were collected and cultured in low-glucose DMEM supplemented with 10% fetal bovine serum (FBS; Gibco), 100 *μ*g/mL penicillin-streptomycin (Sigma, USA), and 2.0 mM glutamine (Sigma) at 37°C and 5% CO_2_. Nonadherent cells were removed 2 days after seeding. At 80-90% confluence, cells were detached with 0.25% trypsin-EDTA and subcultured at a split ratio of 1 : 3 every 2-3 days.

### 2.2. Lentivirus Transduction

hUC-MSCs at passage 3 were seeded in six-well plates at a density of 1 × 10^5^ cells/well. To overexpress or silence c-Jun, lentiviruses carrying either the full-length human c-Jun (Lenti-c-Jun) or a short hairpin RNA (shRNA) targeting human c-Jun (Lenti-shc-Jun) were introduced into hUC-MSCs (MOI, 30) for 12 hours. The c-Jun target sequence for Lenti-shc-Jun was GTGGCACAGCTTAAACAGAAA. The empty vectors (Lenti-NC and Lenti-shNC for Lenti-c-Jun and Lenti-shc-Jun, respectively) served as the control. All lentivirus constructs were labeled with green fluorescent protein (GFP) and were purchased from GeneChem (Shanghai, China). After the transduction was completed, the medium was replaced and the cells were incubated for another 60 hours. The transduction efficiency was assessed by fluorescence microscopy (excitation, 488 nm; emission, 530 nm).

### 2.3. Cell Proliferation Assay

Untransduced cells at passages 5, 10, and 15 and transduced cells at passage 5 were seeded in 96-well plates at a density of 3,000 cells/well and incubated at 37°C for 60 hours. Cell viability was determined using the Cell Counting Kit-8 (CCK-8) assay (Dojindo, Japan) following the manufacturer's instructions.

### 2.4. *In Vitro* Scratch Assay

Cell migration was evaluated with an *in vitro* scratch assay. Untransduced cells at passages 5 and 15 and transduced cells at passage 5 were seeded in six-well plates at a density of 2 × 10^5^ cells/well. The cells were incubated at 37°C for approximately 24 hours until full confluence, and a straight line scratch was made with a 10 *μ*L pipette tip. After the cell debris was removed by rinsing three times with PBS, fresh serum-free medium was applied. The cells were photographed immediately and after 24 hours. The migration of cells to the scratch bed was quantified using ImageJ software.

### 2.5. Animals

Male Sprague-Dawley (SD) rats (6–8 weeks, 200–250 g) were purchased from Hunan SJA Laboratory Animal Co. Ltd. (Changsha, Hunan, China). The rats were housed under pathogen-free conditions in the Department of Laboratory Animals of CSU. All animal studies were approved by the Institutional Animal Care Committee of CSU.

### 2.6. Rat Model of Diabetic Wound Healing

After one-week adaptive feeding, the rats were given a high-sugar and high-fat diet for 4 weeks. The diet consisted of 0.5% sodium cholate, 2% cholesterol, 4% milk powder, 10% fat, 20% sugar, and 63.5% regular diet [10]. Next, the rats received 35 mg/kg streptozocin (STZ) (Sigma) (100 mM solution in citrate-buffered saline at pH 4.5) by intraperitoneal injection. Induction of diabetes was confirmed by a blood glucose test on day 1, day 3, and day 7 after STZ injection (>16.7 mmol/L). The diabetic rats were anesthetized with isoflurane, and the dorsum was shaved. A full-thickness round wound of 1.5 cm diameter was created on the dorsal skin. Afterward, the rats were randomly divided into five groups (four rats per group) and given a total of 200 *μ*L subcutaneous injection of (a) hUC-MSCs, (b) Lenti-NC-transduced hUC-MSCs, (c) Lenti-shc-Jun-transduced hUC-MSCs, (d) Lenti-c-Jun-transduced hUC-MSCs, and (e) PBS, respectively. The wound was divided into four quadrants, and each quadrant received 50 *μ*L injection at the base and edge of the wound. Cell-injected rats received 5 × 10^6^ cells per rat.

The area of the wounds was measured using a Canon PowerShot G9 device on days 1, 3, 7, 10, 15, and 17 of the experiment. The wound edges were drawn, and the wound areas, in pixels, were obtained using the Measure function of ImageJ. The rate of wound closure was calculated using the following equation: wound closure rate (%) = (1 − unhealed wound area/original area) × 100%.

### 2.7. Histologic and Immunohistochemical Analysis

On day 17, the rats were euthanized under isoflurane in a closed chamber after 10-minute exposure to carbon dioxide. The wound bed with surrounding healthy tissues including the epidermis and dermis was immediately harvested, fixed in 10% formalin, and embedded in paraffin. The specimens were sectioned and stained with hematoxylin and eosin (HE) for microscopic analysis of epithelialization. Angiogenesis was evaluated by immunohistochemical analysis of the expression of the endothelial cell marker CD31. In brief, the sections were incubated with a primary anti-CD31 antibody (NB100-64796, 1 : 50 dilution; Novus Biologicals, USA) followed by a biotinylated secondary antibody and horseradish peroxidase- (HRP-) conjugated streptavidin. The immunoreactivity was detected using 3,3-diaminobenzidine and quantified by ImageJ. The sections were counterstained with hematoxylin.

### 2.8. Western Blot Analysis

The cells and wound tissues were lysed in RIPA lysis buffer containing phenylmethanesulfonyl fluoride (PMSF). The lysates were centrifuged at 12,000 g at 4°C for 10 minutes, and the supernatants were subjected to sodium dodecyl sulfate-polyacrylamide gel electrophoresis (SDS-PAGE). The proteins were transferred to polyvinylidene fluoride (PVDF) membranes. After blocking in 5% nonfat milk in PBST for 1 hour at 4°C, the membranes were incubated with primary antibodies toward c-Jun, VEGF, hepatocyte growth factor (HGF), and PDGFA, respectively, at 4°C overnight. All primary antibodies were from Santa Cruz Biotechnology (USA). The membranes were subsequently incubated with HRP-conjugated secondary antibodies, and the protein bands were detected by chemiluminescence.

### 2.9. Real-Time Quantitative Reverse Transcription PCR (qRT-PCR)

Total RNA was extracted with TRIzol reagent (Invitrogen, USA). cDNA was synthesized using the RevertAid RT Reverse Transcription Kit (Thermo Fisher, USA). PCR was performed with the SYBR Green Real-Time PCR Master Mix Kit (Toyobo, Japan) under the following conditions: 5 seconds at 95°C, 15 seconds at 60°C, 40 cycles of 30 seconds at 60°C, and 60 seconds at 72°C. The 2^-*ΔΔ*Ct^ formula was used to calculate the relative gene expression. Data were normalized to *β*-actin. The primers used in PCR were obtained from Sangon Biotech Co. Ltd. (Shanghai, China). The sequences are shown as follows: c-Jun, 5′-GCCTACAGATGAACTCTTTCTGGC-3′ (forward) and 5′-CCTGAAACATCGCACTATCCTTTG-3′ (reverse); *β*-actin, 5′-CTACCTCATGAAGATCCTCACC-3′ (forward) and 5′-AGTTGAAGGTAGTTTCGTGGAT-3′ (reverse).

### 2.10. Statistical Analysis

All data are presented as mean ± standard deviation (SD). Two-tailed Student's *t*-test was used to analyze differences between two groups, and one-way analysis of variance (ANOVA) followed by Tukey's post hoc test for multiple comparison was applied to interpret differences between three or more groups. A *P* value of <0.05 was considered statistically significant.

## 3. Results

### 3.1. *In Vitro* Aged hUC-MSCs Exhibit Reduced Proliferative and Migratory Capacities along with Decreased c-Jun Expression

**Figure 1:**
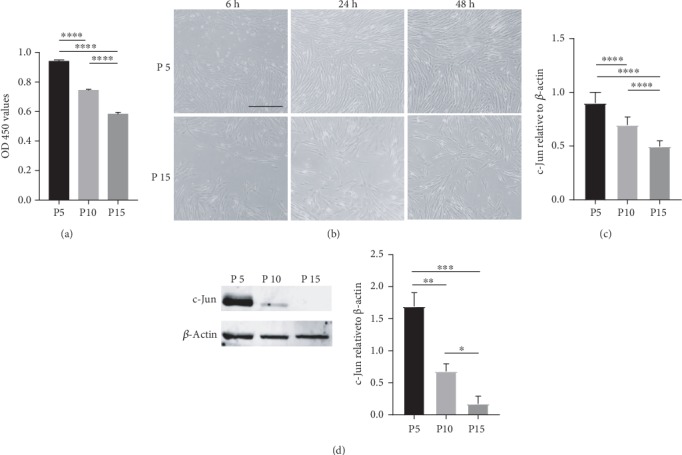
*In vitro* aged hUC-MSCs exhibit reduced proliferative and migratory capacities along with decreased c-Jun expression. (a) The proliferative capacity of hUC-MSCs at passages 5, 10, and 15 determined with the CCK-8 assay. (b) The migratory capacity of hUC-MSCs at passages 5 and 15 evaluated with the *in vitro* scratch assay. (c, d) The relative c-Jun mRNA (c) and protein (d) levels in hUC-MSCs at passages 5, 10, and 15 by qRT-PCR and western blot analysis, respectively. *n* = 4, ∗*P* < 0.05, ∗∗*P* < 0.01, ∗∗∗*P* < 0.001, ∗∗∗∗*P* < 0.0001.

Due to the scarcity and high heterogeneity of freshly isolated MSCs [24], extensive *in vitro* expansion is required to produce sufficient cells for clinical use. However, the stemness and engraftment efficiency of MSCs often decline with increasing passage number [25]. In this study, we observed significant loss in the hUC-MSC proliferative and migratory capacities as the passage number increased from 5 to 15 (Figures 1(a) and 1(b)). Intriguingly, the mRNA and protein expression of c-Jun also declined with increasing passage number (Figures 1(c) and 1(d)), suggesting a potential link between c-Jun and the loss of cell robustness during the *in vitro* aging process.

### 3.2. c-Jun Controls the Proliferative and Migratory Capacities of hUC-MSCs

**Figure 2:**
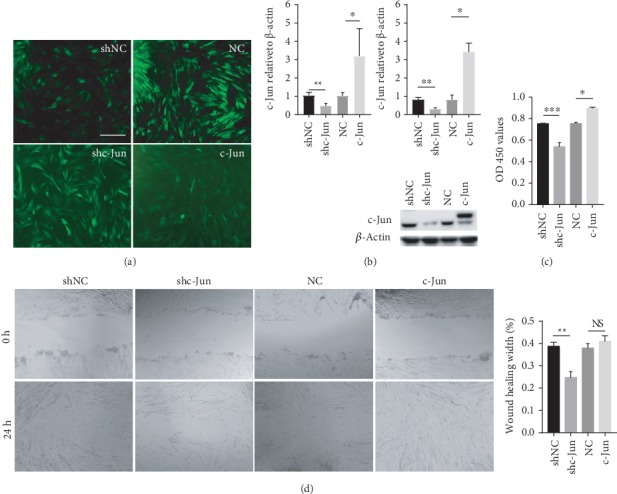
c-Jun controls the proliferative and migratory capacities of hUC-MSCs. hUC-MSCs at passage 3 were transduced with Lenti-NC, Lenti-c-Jun, Lenti-shNC, and Lenti-shc-Jun, respectively. (a) Representative fluorescence cell images are shown (40x magnification). (b) The mRNA (left panel) and protein (right panel) levels of c-Jun determined with qRT-PCR and western blot analysis, respectively. (c) Cell proliferation in the CCK-8 assay. (d) Cell migration in the *in vitro* scratch assay. *n* = 4, ∗*P* < 0.05, ∗∗*P* < 0.01, ∗∗∗*P* < 0.001.

To verify the functional role of c-Jun in the hUC-MSC properties, we transduced hUC-MSCs with Lenti-shc-Jun and Lenti-c-Jun to silence and overexpress c-Jun, respectively. Cells transduced with Lenti-shNC or Lenti-NC served as the controls. Transduction was confirmed to be successful by fluorescence imaging as shown in Figure 2(a). c-Jun silencing and overexpression were confirmed by both qRT-PCR and western blot analysis (Figure 2(b)). We found that c-Jun silencing significantly reduced the proliferative and migratory capacities of hUC-MSCs as indicated by the CCK-8 assay (Figure 2(c)) and the *in vitro* scratch assay (Figure 2(d)), respectively. In contrast, c-Jun overexpression promoted cell proliferation and migration, although the effects on migration did not reach statistical significance (Figures 2(c) and 2(d)). Together, these results suggested that c-Jun positively regulates hUC-MSC proliferation and migration.

### 3.3. c-Jun Promotes Diabetic Would Healing by hUC-MSCs

**Figure 3:**
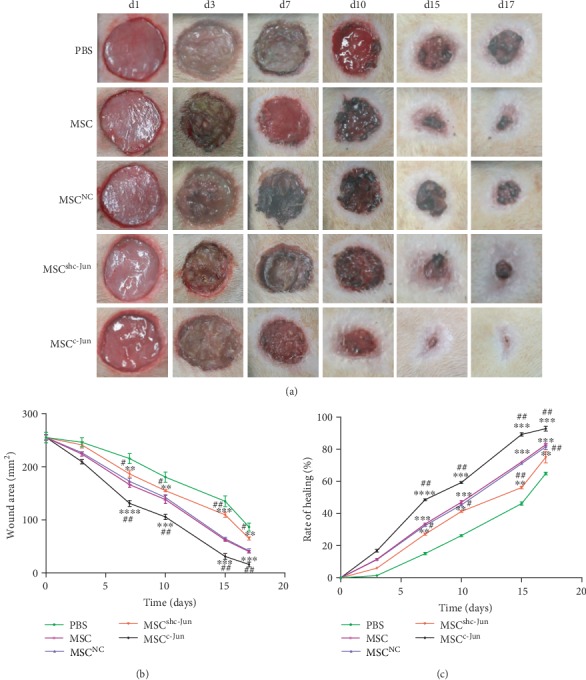
c-Jun promotes diabetic would healing by hUC-MSCs. Diabetic rats received a subcutaneous injection of 5 × 10^6^ hUC − MSCs, untransduced or transduced with Lenti-NC, Lenti-shc-Jun, or Lenti-c-Jun, at the wound edge. PBS served as the control. (a) Representative wound images taken on specific days of the experiment as indicated. (b, c) The wound area (b) and the rate of wound closure (c) on specific days of the experiment as indicated. *n* = 4 rats per group; ∗∗*P* < 0.01, ∗∗∗*P* < 0.001, ∗∗∗∗*P* < 0.0001 vs. PBS; ^#^*P* < 0.05, ^##^*P* < 0.01 vs. hUC-MSC.

**Figure 4:**
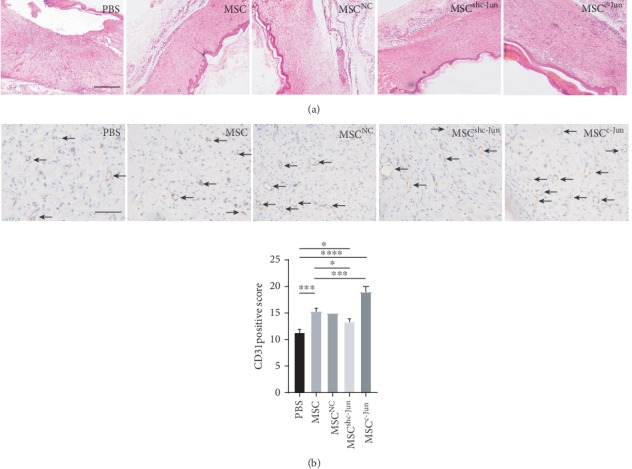
c-Jun accelerates diabetic wound epithelization and angiogenesis by hUC-MSCs. Diabetic rats received subcutaneous injection of 5 × 10^6^ hUC − MSCs, untransduced or transduced with Lenti-NC, Lenti-shc-Jun, or Lenti-c-Jun, at the wound edge. PBS served as the control. The rats were euthanized on day 17 after treatment. (a) Representative images of HE staining of wound tissues (40x magnification). (b) Representative images of immunohistochemical staining for CD31 in wound tissues (200x magnification). *n* = 4, ∗*P* < 0.05, ∗∗∗*P* < 0.001, ∗∗∗∗*P* < 0.0001.

In a rat model of diabetic wound healing, subcutaneous injection of hUC-MSCs (5 × 10^6^ cells per rat) at the wound edge significantly accelerated wound closure compared with the PBS control (Figures 3(a)–3(c)). Compared with untransduced cells, wounds treated with c-Jun-silenced hUC-MSCs showed delayed closure while those treated with c-Jun-overexpressing cells demonstrated expedited healing, with significant differences observed from day 7 (Figures 3(a)–3(c)). Meanwhile, Lenti-NC-transduced hUC-MSCs displayed similar healing efficacy to that of untransduced cells (Figures 3(a)–3(c)). HE staining of the wound tissues revealed more advanced healing as indicated in the greater thickness of the epidermis and dermis in c-Jun-overexpressing wounds compared with untransduced wounds, and the opposite was observed in c-Jun-silenced wounds (Figure 4(a)). Immunohistochemical staining for CD31 showed more advanced angiogenesis in c-Jun-overexpressing wounds compared with untransduced wounds, and the opposite was noticed in c-Jun-silenced wounds (Figure 4(b)).

### 3.4. c-Jun Alters Growth Factor Levels in hUC-MSCs and in Diabetic Wound Tissues

**Figure 5:**
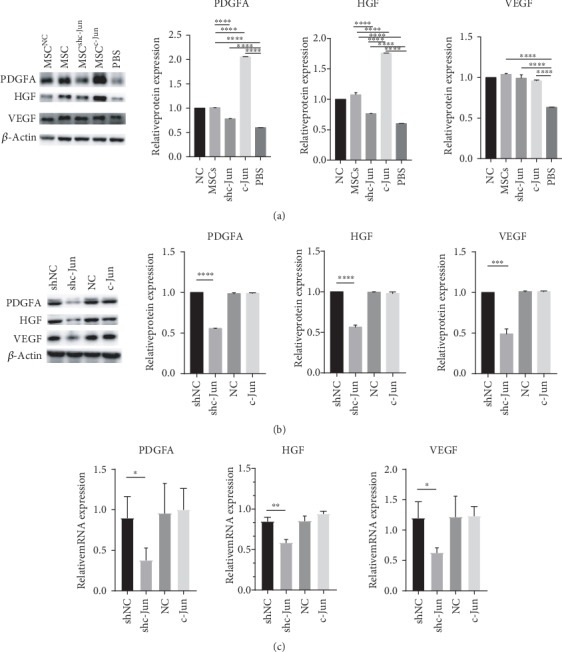
c-Jun alters growth factor levels in hUC-MSCs and in diabetic wound tissues. (a) Diabetic rats received subcutaneous injection of 5 × 10^6^ hUC − MSCs, untransduced or transduced with Lenti-NC, Lenti-shc-Jun, or Lenti-c-Jun, at the wound edge. PBS served as the control. The rats were euthanized on day 17 after treatment. The VEGF, PDGFA, and HGF protein levels in the wound tissues were determined with western blot analysis. (b, c) hUC-MSCs at passage 3 were transduced with Lenti-NC, Lenti-c-Jun, Lenti-shNC, or Lenti-shc-Jun. (b) The VEGF, PDGFA, and HGF protein levels were determined with western blot analysis. (c) The VEGF, PDGFA, and HGF mRNA levels were determined with RT-PCR. *n* = 4, ∗∗∗*P* < 0.001, ∗∗∗∗*P* < 0.0001.

To investigate the mechanisms responsible for the effects of c-Jun, we assessed the levels of growth factors including PDGFA, HGF, and VEGF in dermal and epidermal diabetic wound tissues by western blot analysis. The administration of hUC-MSCs markedly increased the levels of the three growth factors in wound tissues (Figure 5(a)). Compared with tissues treated with untransduced cells, c-Jun-overexpressing cell-treated tissues showed higher PDGFA and HGF levels, while c-Jun-silenced cell-treated tissues exhibited lower levels (Figure 5(a)). However, wound tissues treated with untransduced, c-Jun-overexpressing, and c-Jun-silenced cells all showed similar levels of VEGF (Figure 5(a)). To determine whether these differences in growth factor levels were accounted for by the implanted cells, we evaluated growth factor production in hUC-MSCs prior to implantation. c-Jun silencing markedly reduced the protein and mRNA expression of the three growth factors, while c-Jun overexpression had no significant effects on any of these factors (Figures 5(b) and 5(c)). The differences between the *in vitro* and *in vivo* data suggested that hUC-MSCs may exhibit changed behavior after implantation and/or they may alter growth factor production by other cell types in wound tissues through intercellular communications.

## 4. Discussion

STZ-induced diabetic rats exhibit delayed wound healing than nondiabetic rats [26, 27] and therefore are often used to model impaired wound healing in diabetic patients. hUC-MSCs are commonly used in cell-based therapy because they display robust proliferation and differentiation abilities along with weak immunogenicity [28, 29]. Thus, the benefits of hUC-MSC therapy on wound healing in STZ-induced diabetic rats may be translated to clinical efficacy in human patients.

c-Jun promotes cell cycle progression through regulation of the G1 checkpoint protein cyclin D1 [30] and the G2/M cell cycle kinase Cdk1 [31]. In addition, c-Jun drives cell proliferation by downregulating p53 [32, 33]. c-Jun also regulates the migration of various cell types such as cancer cells during metastasis [34, 35] and epithelial cells during embryogenesis [36]. The wound healing process mainly involves the proliferation and migration of cells, primarily keratinocytes and fibroblasts, and the formation of new blood vessels (angiogenesis); therefore, it is not surprising that c-Jun plays a fundamental role in wound healing [21, 37]. Mice conditionally ablated for c-Jun in the epidermis exhibited deficits in epidermal wound healing, because c-Jun-deficient keratinocytes were unable to migrate or elongate properly [22]. Moreover, the amniotic membrane has been shown to stimulate robust epithelialization in deep wounds by inducing c-Jun expression at the wound border [38]. However, the role of c-Jun in MSC-based therapy for diabetic wound healing is unclear.

hUC-MSCs have been reported to lose proliferative and immunosuppressive capacity during *in vitro* expansion [39]. In the present study, we found that the proliferative and migratory capacities of hUC-MSCs were gradually compromised during *in vitro* expansion, which was accompanied by decreased c-Jun expression. Moreover, c-Jun overexpression in hUC-MSCs accelerated wound closure and increased reepithelization and angiogenesis at the wound bed. Paracrine signaling of growth factors, such as VEGF, PDGF, basic fibroblast growth factor (bFGF), and HGF [40, 41], has been established as mechanisms by which MSCs facilitate tissue repair. Since VEGF, PDGF, and HGF are transcriptionally regulated by c-Jun [42–44], we examined the levels of these growth factors in wound tissues after cell implantation. We detected lower PDGFA and HGF levels in wounds treated with c-Jun-silenced cells and higher levels in those treated with c-Jun-overexpressing cells compared with untransduced cells. However, VEGF levels appeared not to be affected by c-Jun silencing or overexpression. These findings suggested that the enhanced therapeutic effects of c-Jun-overexpressing hUC-MSCs are at least partially accounted for by increased PDGFA and HGF levels in wound tissues. In subcultivated hUC-MSCs, c-Jun silencing decreased intracellular PDGFA, HGF, and VEGF levels, but c-Jun overexpression had no significant effects on any of these growth factors. These differences between *in vivo* and *in vitro* results can be explained by the changed microenvironment for hUC-MSCs after implantation and/or their interactions with other types of cells in the form of paracrine signaling, which is only possible *in vivo* but not *in vitro*. One of the limitations of the *in vitro* study was that we did not examine the growth factor levels in the culture medium, which would indicate the secretion of these proteins.

## 5. Conclusion

*In vitro* expansion of hUC-MSCs resulted in the loss of proliferative and migratory capacities accompanied by decreased c-Jun expression. c-Jun overexpression promoted hUC-MSC proliferation and migration *in vitro* and accelerated diabetic wound closure, reepithelization, and angiogenesis by hUC-MSCs *in vivo*. These beneficial effects of c-Jun overexpression in diabetic wound healing were at least partially mediated by increased PDGFA and HGF levels in wound tissues.

## Data Availability

The data used to support the findings of this study are available from the corresponding author upon request.

## Supplementary Materials

Supplementary Materials The blood glucose levels of the animals during the experiment showing STZ induction.
