# Supplementary material for: c-Jun Overexpression Accelerates Wound Healing in Diabetic Rats by Human Umbilical Cord-Derived Mesenchymal Stem Cells
Source: Stem Cells Int. 2020 Jan 14;2020:7430968. doi: 10.1155/2020/7430968 (PMC7201444; doi:10.1155/2020/7430968)
Supplement: Supplementary Materials — The blood glucose levels of the animals during the experiment showing STZ induction. [file 7430968.f1.pdf]

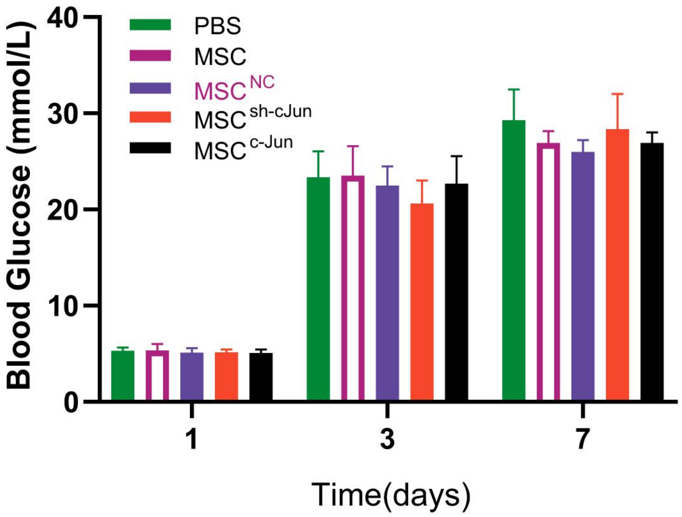

supplementary figure

The blood glucose levels of the animals during the experiment showing STZ-induced hyperglycemia in the diabetic rat model.
